# Yu Ping Feng San Exert Anti-Angiogenesis Effects through the Inhibition of TSLP-STAT3 Signaling Pathways in Hepatocellular Carcinoma

**DOI:** 10.1155/2019/1947156

**Published:** 2019-10-23

**Authors:** Qin Yuan, Fei Yao, Liang Zhou, Guoqiang Liang, Xiudao Song, Guorong Jiang, Man Zhou, Lurong Zhang

**Affiliations:** ^1^Suzhou TCM Hospital Affiliated to Nanjing University of Chinese Medicine, Suzhou 215000, China; ^2^Suzhou Academy of Wumen Chinese Medicine, Suzhou 215000, China

## Abstract

**Background:**

Clinically, Yu ping feng san (YPFS) has been extensively used as a medication for treating immune deficiency, and YPFS is combined with chemotherapy drugs to treat cancer, including hepatocellular carcinoma (HCC), lung cancer, and pancreatic cancer. Previous research has shown that YPFS has a therapeutic effect on HCC by improving the immunosuppressive state of the liver cancer microenvironment. The present study aimed to investigate the effect of YPFS on angiogenesis of HCC.

**Methods:**

High-performance liquid chromatography (HPLC) was used to certify the composition of YPFS. An orthotopic transplanted model of murine HCC was entrenched. Immunohistochemistry was used to observe the changes of the microvessel density (MVD). The MTT assay was used to detect the cell viability. ELISA was performed to analyze the expression of related factors. Western blot was used to analyze the protein expression. Tube formation assay was used to analyze the anti-angiogenic efficiency.

**Results:**

YPFS significantly reduced the tumor volume and weight, thus exerted the growth inhibitory effect. The level of MVD and VEGF was obviously decreased in YPFS-treated HCC-bearing mice, and the YPFS treatment also reduced the VEGF level in Hepa1-6 cells. Further study revealed that the expression of TSLP/TSLPR and p-STAT3/STAT3 was decreased by YPFS. The level of MVD and VEGF and the expression of TSLP/TSLPR and p-STAT3/STAT3 in tumor tissue and Hepa1-6 cells were suppressed by incubation with the anti-TSLP antibody, whereas treatment with the anti-TSLP antibody in YPFS-treated cells did not cause further significant inhibition compared with the cells treated only with YPFS. More importantly, YPFS inhibited proliferation, expression of p-STAT3/STAT3, and tube formation of HUVECs induced by TSLP.

**Conclusions:**

These results indicated that YPFS attenuated the activation of the TSLP-STAT3 signaling pathway by inhibiting the immune-related factor-TSLP, thereby inhibiting the formation of hepatic microvessels and exerting an anti-HCC effect.

## 1. Introduction

Hepatocellular carcinoma (HCC) is a primary liver cancer, the third leading cause of cancer mortality worldwide [1]. It has high malignancy, strong invasiveness, and poor prognosis; as a consequence, many chemotherapy agents are of limited use and provide minimal benefit to survival time in HCC patients. [2]. There is an invariable need to research more competent therapeutic strategies for HCC patients. We all know that angiogenesis exists in solid tumors and performs an indispensable role in affording nutrient substances for tumor cell proliferation, invasion, and metastasis [3]. HCC is a hypervascular carcinoma, and the excessive expression of angiogenic factors will lead to uneven distribution of blood and oxygen in liver cancer tissues and increases the local infiltration of tumor microvessels, leading to enhancing growth and metastasis of liver cancer [4]. Therefore, suppressing neovascularization to diminish the recurrence and metastasis of tumor has become one of the efficient methods for the treatment of HCC. However, anti-angiogenic therapy alone is not sufficient for bettering patient survival in clinical treatment, overpowering resistance, and generating enduring clinical responses in HCC; thus, anti-angiogenic drugs are generally used in combination with immune-enhancing or chemotherapeutic agents [5].

The recognition that tumor possesses a feature of the inflammatory microenvironment and immune disease is the most remarkable achievement in recent years. Pro-inflammatory cytokines in the tumor microenvironment are known for their ability to benefit cancer evolution via an angiogenic effect [6]. The abnormal tumor vasculature, induced by the production of pro-angiogenic factors, sustains an immunosuppressive tumor microenvironment, intensifying the tumor potential to avoid the host immunosurveillance [7, 8]. Researches show that improving tumor immune suppression microenvironment has an important effect on the inhibition of tumor microvasculature in preventing the occurrence and development of HCC [9]. Furthermore, anti-angiogenic therapy alone is not sufficient for improving immunosuppression, but its treatment effect is also not ideal. Therefore, the recognition of an anti-angiogenic lead compound with immune-enhancing efficacy and few side effects that may serve as a natural chemopreventive agent is becoming progressively important [10].

Thymic stromal lymphopoietin (TSLP) is an inflammation associated IL-7-like cytokine. It primarily required signaling through the TSLP receptor on CD4 + T cells, promoting Th2-skewed immune responses to make the body under the immunosuppressive , thus inducing occurrence and development of the disease [11, 12]. In recent years, a large number of studies have shown that TSLP is highly expressed in a variety of tumors, including liver cancer, breast cancer, melanoma, etc., and is negatively correlated with the survival time of patients [13]. The expression of TSLP is significantly associated with tumor development and metastasis; knockout TSLP expression can almost completely prevent cancer progression and lung metastasis [14]. TSLP secreted by cancer cells promotes the development and metastasis of cancer by inducing the expression of vascular endothelial growth factor (VEGF) [15]. It was found that TSLP functions mainly through the JAK/STATs signal transduction pathway, and TSLP can induce phosphorylation of all known STATs except STAT2 [16]. Meanwhile, the study demonstrated that STAT3 is a direct transcriptional activator of VEGF, and inhibition of STAT3 activation by STAT3 negative mutant can significantly inhibit the VEGF expression and angiogenesis, thereby limiting the tumor growth and metastasis [17, 18]. It has been confirmed in mouse implanted with human HCC that inhibition of STAT3 activation by antisense oligonucleic acid not only reduces the expression of VEGF and MMP-2 but also reduces the microvascular density (MVD) of CD34 positive in tumor tissues, inhibiting tumor metastasis to the liver [19]. Therefore, TSLP-STAT3 signaling pathway may play a key role in the neovascularization of HCC.

Yu ping feng san (YPFS), originally recorded in the book Danxi Xinfa, is an ancient Chinese medical classic formula; it has been used in clinical treatment for more than six hundred years, consisting of Astragali Radix (AR; Huangqi; the root of *Astragalus membranaceus* (Fisch.) Bunge or *Astragalus membranaceus* (Fisch.) Bunge var. *mongholicus* (Bunge) P.K. Hsiao), Atractylodis Macrocephalae Rhizoma (AMR; Baizhu; the rhizomes of *Atractylodes macrocephala* Koidz.), and Saposhnikoviae Radix (SR; Fangfeng; the roots of *Saposhnikovia divaricata* (Turcz.) Schischk.). Clinically, YPFS has been shown to produce beneficial immune-modulatory effects of preventing bacterial and viral infections [20] and was widely used as medication for the treatment of respiratory and immune system diseases, including anaphylactic rhinitis, asthma, and COPD [21, 22]. In cancer therapy, treatment with YPFS showed an enhanced effect on the anti-tumor immune responses of patients with primary liver cancer [23]. Many recent studies reported that YPFS could increase the immune function to inhibit tumor growth and metastasis [24–26]. Our previous research indicated that YPFS has a therapeutic effect on HCC by improving the immunosuppressive state of the liver cancer microenvironment and has no toxicity. Meanwhile, we also found YPFS could significantly reduce the expression of the TSLP in tumor and adjacent tissues [27–29]. However, whether YPFS regulates the immune-related factor TSLP to attenuate the activation of the TSLP-STAT3 signaling pathway, thereby inhibits the formation of angiogenesis and exerts an anti-HCC effect remain unknown. Therefore, this study aimed to assess the anticancer effect of YPFS on human HCC cells in vivo and in vitro. Moreover, we aimed to elucidate its potential molecular mechanisms.

## 2. Materials and Methods

### 2.1. Preparation of YPFS and Determination of Effective Content

The TCM formula in our study was YPFS, which composed of three herbs: the roots of *A. membranaceus var. mongholicus* (AR), the rhizomes of *A. macrocephala* (AMR), and the roots of *S. divaricata* (SR). All herbs of YPFS were purchased from Chunhui Tang Pharmaceutical Co., Ltd (Suzhou, China). The identification of herbs was according to the standards of Astragali Radix, Atractylodis Macrocephalae Rhizoma, and Saposhnikoviae Radix of the Chinese Pharmacopoeia (Part 1, 2015 Edition) by Dr. Lurong Zhang. The herbal decoction was prepared using methods as follows: typically, according to the Danxi Xinfa prescription, we weighted the crude materials (in slices) 50 g AR, 150 g AMR, and 50 g SR, the herbal mixture (AR : AMR : SR in a 1 : 3 : 1 weight ratio). We added 3 times of distilled water (750 mL), soaked for 0.5 hour; in addition, added 5 times of water (1250 mL), refluxed for 1.5 hours (100°C), filtered and collected the filtrate. The drug residue was further mixed with 6 times of water (1500 mL), and refluxed for 1 hour (100°C), and the filtrate was combined twice. The filtrate was concentrated in an appropriate amount to obtain an extract, frozen at −20°C overnight, and lyophilized to powder. The weighted result was noted: 250 g crude herbs got 114.2 g powder; the yield was 45.6%. 20, 30, and 40 g crude herbs/kg (abbreviation: 20, 30, and 40 g/kg) YPFS powder solution, according to the yield of the drug, weighing a certain amount of YPFS powder in distilled water.

To characterize the active ingredients of YPFS, high-performance liquid chromatography (HPLC) was used. The separation was carried out in Hypersil ODS column (250 mm *∗* 4.0 mm, 5 *μ*m) by gradient elution with acetonitrile-water mixtures. The mobile phase elution programme is reported in Table 1. The flow rate was 1.0 mL/min, the detection wavelength was 220 nm, and the column temperature was 30°C. The injection volume was 5 *μ*L.

Using this technique, the reference fingerprint and fifteen common peaks from the HPLC fingerprint of YPFS decoction were analyzed (Supplementary [Supplementary-material supplementary-material-1]). The compounds of nine peaks were identified, and the contents were analyzed (Table 2).

### 2.2. Cell Culture and Cell Viability Assay (MTT Assay)

The murine HCC Hepa1-6 cell line was purchased from Cell Bank of the Chinese Academy of Sciences (Shanghai, China); the catalogue code was SCSP-512. Cells were grown in a high-glucose Dulbecco's modified eagle's medium (DMEM; Gibco, cat. no. 1416723), supplemented with 10% heat-inactivated fetal bovine serum (Hangzhou Sijiqing Biological Engineering Materials Co., Ltd. cat. no. 140902) at 37°C in a humidified atmosphere containing 5% CO_2_. Then, we treated the cell with YPFS, low dose was 10 mg crude herbs/mL (abbreviation: 10 mg/mL), median dose was 30 mg crude herbs/mL (abbreviation: 30 mg/mL), and high dose was 50 mg crude herbs/mL (abbreviation: 50 mg/mL). Human umbilical vein ECs (HUVECs) was purchased from Beina Biological Co., Ltd (Suzhou, China); the registration number is 08795844440321. Cell lines were routinely cultured in a DMEM supplemented with 10% fetal bovine serum at 37°C in a humidified atmosphere of 5% CO_2_.

The cell viability of HUVEC treated with YPFS (the low dose was 10 mg/mL, the median dose was 30 mg/mL, and the high dose was 50 mg/mL) and TSLP (100 ng/mL) was investigated using the modified MTT assay. HUVECs (1 *∗* 104 cells/well) were seeded in 96-well plates in triplicates with 100 *μ*L medium per well. After overnight incubation at 37°C, the cells were then treated with indicated concentrations of YPFS and TSLP for 48 hours. Subsequently, 20 *μ*L of MTT solution (5 mg/mL in PBS) was added to each well, incubation was continued for 4 hours, the culture was terminated, and the culture supernatant in the well was carefully aspirated. 150 *μ*L of DMSO was added to each well and shaken for 10 minutes to fully melt the crystal. Colorimetric: the selected wavelength was 570 nm, then the light absorption value of each hole on the enzyme-linked immunoassay monitor was determined, and the result was recorded. Three independent experiments were performed.

### 2.3. Animal Model and Treatment: Assessment of Tumor Weight

The male C57/BL6 mice (*n*, 32; age, 6–8 weeks; weight, 20–22 g; purchased from the animal experiment center of Matt Albert Technology Co., Ltd., Suzhou, China; animal certificate no. SCXK (JING) 2014-0004) were used for the formulation of a mouse model of HCC ensuring flexible feeding for 3 days.

We built an orthotopic transplanted model of murine HCC according to the previous studies [30]. After administering abdominal anesthesia in mice, the supine position was taken, and the limbs were fixed on the experimental plate. The body hair was removed with 10% sodium sulfide. After disinfection with anil iodine, the abdominal abdomen was opened layer by layer, the abdominal cavity of the mice was exposed, and the mice were gently compressed. In the chest, the liver jumped out of the abdomen, and the liver closest to the body surface was selected, to grow the tumor. Hepa1-6 cell suspension was prepared by using an oblique needle. Placed the needle tip such that the needle tip and the horizontal plane are 20 degrees into the liver about 1 cm, gently pushed the syringe needle, slowly injected the cell suspension 0.05 mL, about 1 *∗* 10^6^ cells of Hepa1-6. Gradually, the needle was withdrawn, and gently pressed on a sterile cotton swab for a while, then smoothly delivered the liver to the abdominal cavity, and close the abdomen layer by layer. Ten days after the model was created, a laparotomy was performed to determine the tumor formation in the liver, and the tumor size was measured for group administration. Animals were housed in a laminar flow cabinet free of pathogens, and small animal diagnostic ultrasound was performed early and late to determine the presence of tumors and to assess tumor burden.

The grouping method we experimented with was stratified random grouping. Tumor-bearing mice were randomly assigned to eight subgroups according to their body weight. Each subgroup contained four identical mice and then one mouse was randomly selected from each subgroup to be assigned to the experimental groups, including the vehicle group (saline group) and the YPFS three doses (20, 30, and 40 g crude herbs/kg, respectively). The YPFS dose for mice was calculated from the adult daily dose recommended in the Chinese Pharmacopoeia 2015 version. So that, each experimental group contains 8 mice. Dosing began on the day of grouping. The vehicle group was given distilled water once a day, and the YPFS group was administered the drug once a day. After the last administration, the mice were fasted for 12 hours, then were anesthetized by intraperitoneal injection of 50 mg/kg pentobarbital sodium (Sigma-Aldrich; Merck KGaA), and the anesthetized mice were sacrificed by CO2 exposure (displacement rate, 20%/min). All experimental protocols and procedures are carried out in accordance with the EU Directive 2010/63/EU Animal Experiment [31]. This study was permitted by the Ethics Committee of Suzhou Chinese Medicine Hospital (Suzhou, China).

The tumor tissues were weighed and collected. Tumor inhibition rate (%) = (mean tumor weight of the vehicle group − mean tumor weight of experiment group)/mean tumor weight of vehicle group × 100%.

### 2.4. Immunohistochemistry (IHC) Assay and Analysis

The paraffin-embedded liver tumor tissue was cut into 4 *μ*m, fixed in 4% formalin for 24 hours at 25°C, dewaxed, sectioned in xylene for 3 washes, washed twice in 100% ethanol, washed twice in 95% ethanol, and washed twice in ddH_2_O. To extract the antigen, the slide was placed in 10 mM sodium citrate buffer, the sub-boiling point was maintained for 10 minutes, and cooled for 30 minutes. Sections were incubated in 3% hydrogen peroxide for 10 min to block endogenous peroxidase and were washed in ddH2O twice for 5 min each, and blocked each section with 100‑150 µl blocking solution (1% w/v BSA,TBS) for 2 h at 25˚C. The sections were subjected to staining procedures with purified monoclonal anti-rabbit CD34primary antibody (dilution, 1:250; cat. no. ab81289; Abcam, Cambridge, UK) at 4˚C overnight. Subsequently, the biotinylated goat anti-rabbit immunoglobulin secondary antibody (dilution, 1 : 2,000; cat. no. ab205718; Abcam, Cambridge, UK) was incubated for 1 hour at 25°C. Then, rabbit-specific HRP conjugate (cat. no. D110117; Sangon Biotech Co., Ltd., Shanghai, China) was used for 15 minutes at 25°C. The sections were then counterstained with hematoxylin for 1 to 2 minutes at 25°C, dehydrated by using ethanol and xylene. Immunohistochemistry results were quantified using Olympus DP72 software and Image-Pro Plus 6.0 analysis software. Ten different areas for each area in the random area were selected. The microvessel density (MVD) was determined as described above. The area with the highest degree of vascularization was identified and examined under a 100x microscope, and one area of each of the ten vascularized areas was calculated under a 200x microscope. The average of the ten regions was recorded as the MVD level of the slide. Results were compared as a percentage of the treatment group. All sections were independently analyzed by two double-blind pathologists and reconfirmed when the results were inconsistent.

### 2.5. ELISA Assays of the Expression of VEGF and TSLP/TSLPR

Tumor tissue samples of liver cancer mice treated with saline or YPFS were placed on ice with the RIPA buffer (10 mM Tris-HCl pH 7.5, 120 mM NaCl, 1% NP-40, 1% sodium deoxycholate, and 0.1% SDS). After centrifugation at 2000 ×g for 5 minutes at 4°C, the supernatant (100 *μ*L) was taken for analysis; the level of VEGF in the tissue homogenate was quantified according to a commercially available ELISA kit (cat. no. 110518007; eBioscience; Thermo Fisher Scientific, Inc.) according to the manufacturer's instructions. Quantitative determination of TSLP/TSLPR concentration in tissue homogenate by commercially available ELISA kit (cat. no. 117921642; eBioscience; Thermo Fisher Scientific, Inc).

### 2.6. Western Blot Analysis

Liver tissues extracts were prepared from liver cancer tissue specimens and the cell protein from HUVEC treated with the RIPA buffer. Then, an equal amount of protein (50 *μ*g) was loaded onto 10% SDS-PAGE and transferred onto polyvinylidene difluoride membranes. The membranes were blocked with 5% skimmed dried milk buffered for 2 hours at 25°C and incubated with primary antibodies against STAT3 rabbit mAb (dilution, 1 : 1,000; cat. no. 9959; Cell Signaling Technology, Inc., Danvers, MA, USA), p-STAT3 antibody (dilution, 1 : 1,000; cat. no. 9914; Cell Signaling Technology, Inc.), and PCNA antibody (dilution, 1 : 1,000; cat. no. 2586; Cell Signaling Technology, Inc.), overnight at 4°C. Next, the membranes were washed and incubated with horseradish peroxidase-conjugated secondary antibody for 2 hours at 25°C. The membranes were washed four times with tris-buffered saline with Tween-20 and detected by a fluorescence visible imaging system BIO-RAD imaging system (BIO-RAD, Hercules, CA, USA). The intensity of each band was evaluated by the densitometry test using Image Pro Plus 4.5 software (Media Cybernetics, Inc., Bethesda, MD, USA). The quantification was normalized to the comparable value of GAPDH expression.

### 2.7. Tube Formation Assay

The day before the experiment, 20 *μ*L and 1 mL tips and 48-well plates in a 4°C refrigerator was prepared. Matrigel™ (50 *μ*L; cat. no. 356230; BD Bedford, MA, USA) was 4 degrees frozen for 2 hours and placed in an ice bath when the matrigel was in a liquid state. The matrix gel was diluted 3 times with a DMEM, and 150 *μ*L matrigel-medium suspension was added to each well of the 48-well plate; the whole process was carried out in an ice bath. The treated plate was placed in a 37°C incubator for about 30 minutes. Inoculation of cells and administration were done as follows: HUVEC were digested in a logarithmic growth phase, centrifugated, and adjusted the cell density to 2 *∗* 10^4^, 200 *μ*L of medium per well. Except for the control group, the other groups were treated with various concentrations of YPFS (10, 30, and 50 mg/mL) for 24 hours and resuspended in a DMEM with 10% FBS with or without TSLP (100 ng/mL). After approximately 6–10 hours, the tube began to form, and the images were obtained using the Nikon Ti-U inverted fluorescence microscope.

### 2.8. Statistical Analysis

All experiments were repeated at least three times, and the results of the experimental studies were expressed as the mean ± the standard error of the mean. Differences between the different treatment groups were assessed by ANOVA followed by Dunnett's multiple comparison posttest (GraphPad Prism 5 Software, San Diego, CA, USA). A *P*-value of 0.05 was considered as an indication for statistical significance.

## 3. Results

### 3.1. Inhibitory Effects of YPFS on HCC-Bearing Mice

**Figure 1:**
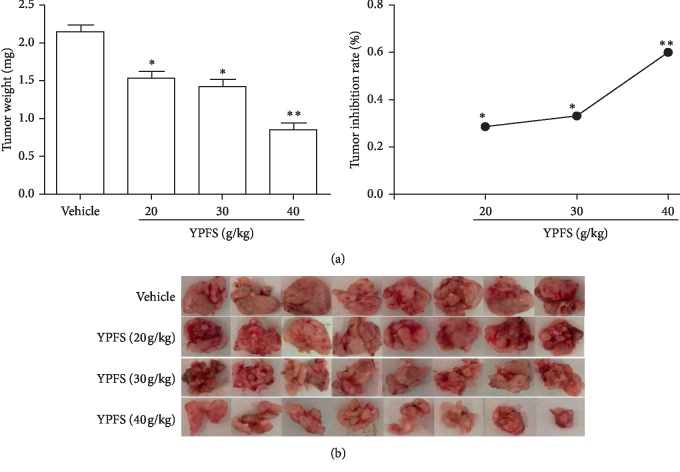
Inhibitory effects of YPFS in HCC-bearing mice. (a) Tumor weights of the HCC-bearing mice treated with or without YPFS. Calculating the tumor inhibition treated with different concentrations of YPFS. (b) Images of final excised tumors. All plotted values are means ± SD (*n* = 8). Significance: ^*∗*^*P* < 0.05, ^*∗∗*^*P* < 0.01 compared with the vehicle group.

We injected Hepa1-6 cells into the left hepatic lobe of mice and successfully established an HCC mouse model. We examined the effect of YPFS on the growth of tumors in HCC-bearing mice. The tumor weights of YPFS-treated mice (20, 30, and 40 g/kg) were significantly decreased compared with those of the vehicle group, and the tumor growth inhibition ratio of the YPFS-treated mice (20, 30, and 40 g/kg) was obviously increased compared with vehicle group mice in a dose-dependent manner (*P* < 0.05 and *P* < 0.01; Figure 1(a)). The tumor was oval after resection, the surface was smooth, the boundary was clear, and the capillary network was rich, the tumor from YPFS-treated mice (20, 30, and 40 g/kg) exhibited a decreasing trend (Figure 1(b)). Taken together, these results demonstrated that YPFS inhibited the tumor growth of HCC.

### 3.2. Effects of YPFS on the Angiogenesis of HCC

**Figure 2:**
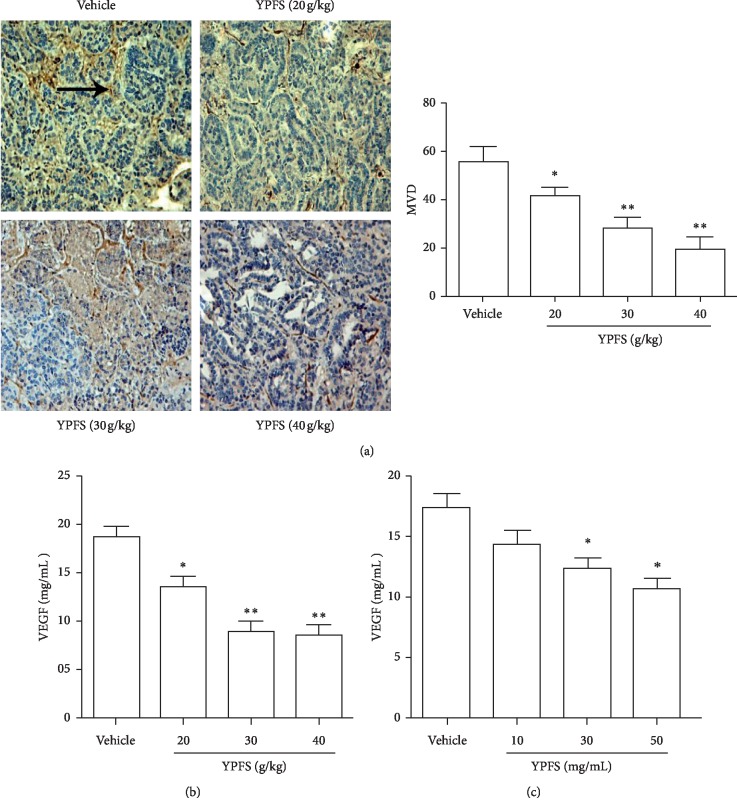
Effects of YPFS on the angiogenesis of HCC. (a) Tumor tissues from HCC-bearing mice treated with indicated concentration of YPFS (20, 30, and 40 g/kg) were performed by immunohistochemical staining for detecting vascular marker CD34. The pointed tip indicated a positive result and was observed under a 200x microscope. (b) Expression levels of VEGF in tumor tissues were measured by ELISA. (c) Expression levels of VEGF in Hepa1-6 cell were measured by ELISA. Data are presented as the mean ± standard error of the mean. The tumor tissues were observed with a light microscope at ×200 magnification. ^*∗*^*P* < 0.05, ^*∗∗*^*P* < 0.01, compared with the vehicle group.

To systematically assess the mechanism of anti-tumor activity of YPFS, we first evaluated its effects on angiogenesis of HCC *in vivo*, MVD and VEGF are critical factors involved in the ability of tumor tissue to induce angiogenesis [32]. CD34 is considered to be a sensitive and well-defined marker of hepatic microvessels. In this study, the expression of CD34 in microvessels was used as a surrogate marker to detect the expression of MVD in tissues. IHC for CD34-positive microvessels expression was performed on tumor tissue. Compared with the vehicle group, MVD in the tumor tissues of the YPFS treatment groups was significantly decreased (*P* < 0.05 and *P* < 0.01; Figure 2(a)). To more closely examine the anti-angiogenic effects of YPFS, we subsequently examined the expression of VEGF in tumor tissue by using ELISA. Compared with the vehicle group, VEGF in the tumor tissues in response to YPFS treatment was significantly decreased in a dose-dependent manner (*P* < 0.05 and *P* < 0.01; Figure 2(b)). These results showed that YPFS inhibited the angiogenesis of HCC *in vivo*. Then, we examined the effects of YPFS on the secretion of VEGF in Hepa1-6 cells. As shown in Figure 2(c), the expression of VEGF was markedly decreased in response to YPFS in a dose-dependent manner. The results show that YPFS inhibited the expression of VEGF in Hepa1-6 cells.

### 3.3. Effects of YPFS on the Signal Pathways of TSLP/STAT3

**Figure 3:**
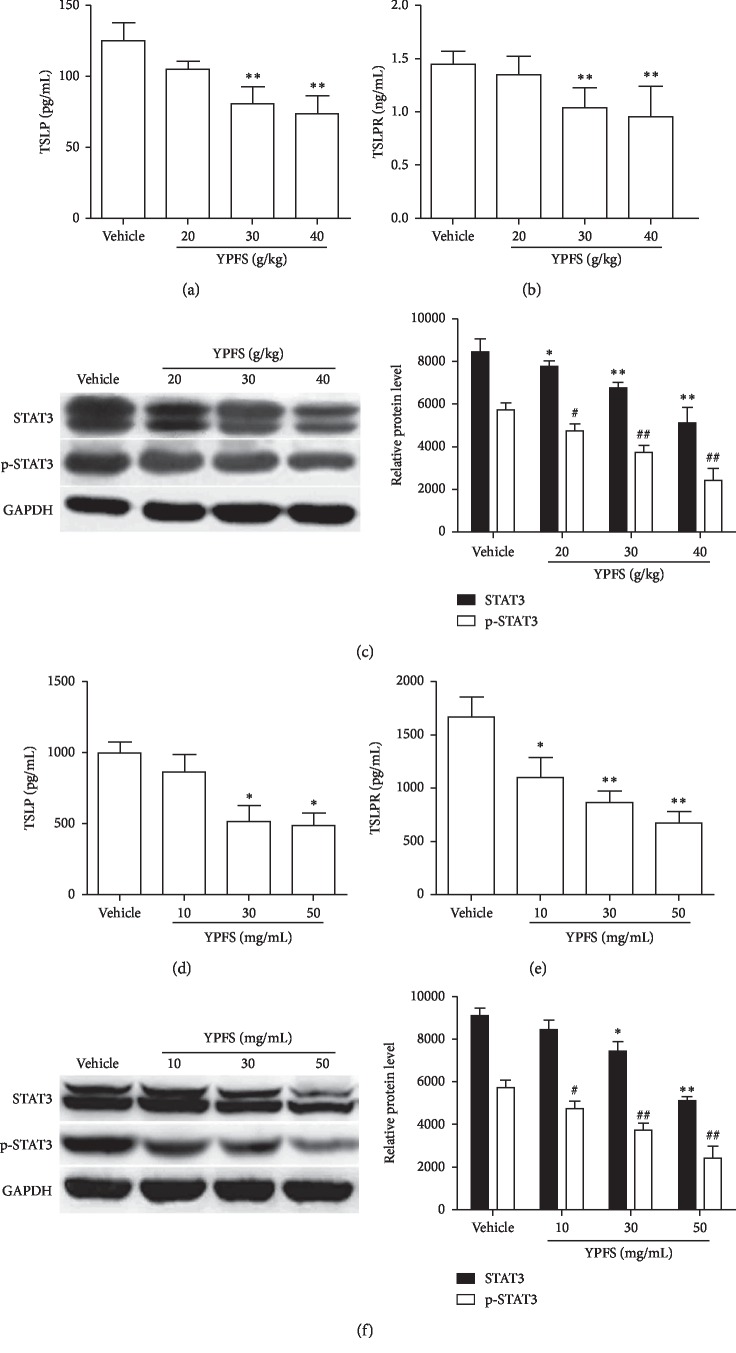
Effects of YPFS on the signal pathways of TSLP/STAT3. (a) Expression levels of TSLP in tumor tissues treated with indicated concentration of YPFS were measured by ELISA. (b) Expression levels of TSLPR in tumor tissues treated with indicated concentration of YPFS were measured by ELISA. (c) The expression of STAT3 and p-STAT3 in tumor tissues treated with indicated concentration of YPFS detected by western blot. (d) ELISA analyzed the expression of TSLP in Hepa1-6 cell of the YPFS treatment. (e) ELISA analyzed the expression of TSLPR in Hepa1-6 cell of the YPFS treatment. (f) The expression of STAT3 and p-STAT3 in Hepa1-6 cell of the YPFS treatment detected by western blot. Quantification of western blot analysis of p-STAT3 and STAT3 levels normalized by the levels of GAPDH. Data are presented as the mean ± standard error of the mean. ^*∗*^*P* < 0.05, ^*∗∗*^*P* < 0.01, ^#^*P* < 0.05, ^##^*P* < 0.01 compared with the vehicle group.

Analyses of expression profiles of possible target proteins are helpful for us to understand the roles of the signaling molecules involved the mechanisms of anti-angiogenesis of YPFS. Studies have reported that TSLP and TSLPR expression in the tumor cells contributes to tumor growth following tumor microenvironment Th2 cell differentiation [11]. To determine whether the expression of TSLP induced by YPFS was responsible for the inhibition of angiogenesis, we detected the expression of TSLP and TSLPR by ELISA in tumor tissue of HCC-bearing mice treated with YPFS. Compared with the vehicle group, the expression of TSLP and TSLPR of the YPFS treatment groups was obviously decreased in a dose-dependent manner (*P* < 0.01; Figures 3(a) and 3(b)). It was found that TSLP functions mainly through the JAK/STATs signal transduction pathway, and TSLP can induce phosphorylation of STAT3 at tyrosine 705 (Tyr705). Meanwhile, the study demonstrated that STAT3 is a direct transcriptional activator of VEGF. Thus, we next investigated whether YPFS affected STAT3 activation in HCC-bearing mice. We found that compared with the vehicle group, the total STAT3 and p-STAT3 protein expression was significantly reduced in a dose-dependent manner in the YPFS-treated groups compared with the vehicle group (*P* < 0.05 and *P* < 0.01; Figure 3(c)), These results revealed that YPFS exerts the anti-angiogenic activity *in vivo* through inhibiting the activation of TSLP/STAT3 signaling pathway. Furthermore, we examined whether the similar results were observed in YPFS-treated Hepa1-6 cell *in vitro*. We also found that compared with the vehicle group, the expression of TSLP and TSLPR in Hepa1-6 cell of the YPFS-treated groups exhibited a decreasing trend (Figures 3(d) and 3(e)). Similarly, the expression of total STAT3 and p-STAT3 was significantly reduced in a dose-dependent manner in YPFS-treated groups (*P* < 0.05 and *P* < 0.01; Figure 3(f)). These results showed that YPFS inhibited the expression of TSLP/TSLPR and STAT3/p-STAT3 in HCC tumor tissue and tumor cells. And, it is suggested that YPFS may exert its inhibitory effect on angiogenesis by regulating TSLP/STAT3 pathway.

### 3.4. Anti-TSLP Antibody Can Attenuate the Inhibitory Effect of YPFS on Angiogenesis and VEGF Secretion

**Figure 4:**
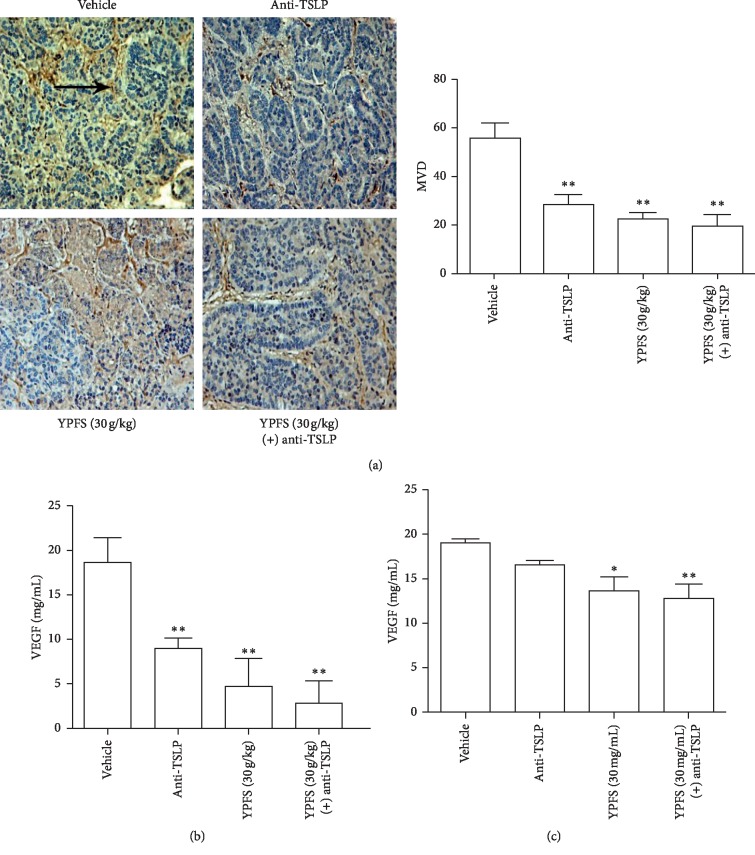
Anti-TSLP antibody can attenuate the inhibitory effect of YPFS on angiogenesis and VEGF secretion. (a) Tumor tissues treated with the anti-TSLP antibody and indicated concentration of YPFS (30 g/kg) mice were performed by immunohistochemical staining for detecting vascular marker CD34. The pointed tip indicated a positive result and was observed under a 200x microscope. (b) Expression levels of VEGF in tumor tissues treated with the anti-TSLP antibody and indicated concentration of YPFS (30 g/kg) mice were measured by ELISA. (c) Expression levels of VEGF in Hepa1-6 cell treated with the anti-TSLP antibody and indicated concentration of YPFS (30 mg/mL) mice were measured by ELISA. The tumor tissues were observed with a light microscope at ×200 magnification. Data are presented as the mean ± standard error of the mean. ^*∗*^*P* < 0.05, ^*∗∗*^*P* < 0.01, compared with the vehicle group.

To probe into whether YPFS inhibited the tumor angiogenesis through affecting the activation of TSLP, we used the anti-TSLP antibody to detect the level of MVD and VEGF in tumor tissues. We found MVD in the tumor tissues treated with the anti-TSLP antibody was significantly decreased compared with the vehicle group; however, MVD in the HCC tissues treated with the anti-TSLP antibody and YPFS did not cause further significant inhibition compared with the group treated with YPFS only (*P* < 0.01; Figure 4(a)), and the expression changed VEGF in accordance with the MVD (*P* < 0.01; Figure 4(b)). Then, we also examined the level of VEGF in Hepa1-6 cell, and we found the results were consistent with the results in vivo (*P* < 0.05 and *P* < 0.01; Figure 4(c)). These results suggested that YPFS has a potential target effect on the inhibition of the activation of TSLP; inhibiting the activation of TSLP could suppress angiogenesis of HCC.

### 3.5. Anti-TSLP Antibody Can Attenuate the Inhibitory Effect of YPFS on the Signal Pathways of TSLP/STAT3 in HCC

**Figure 5:**
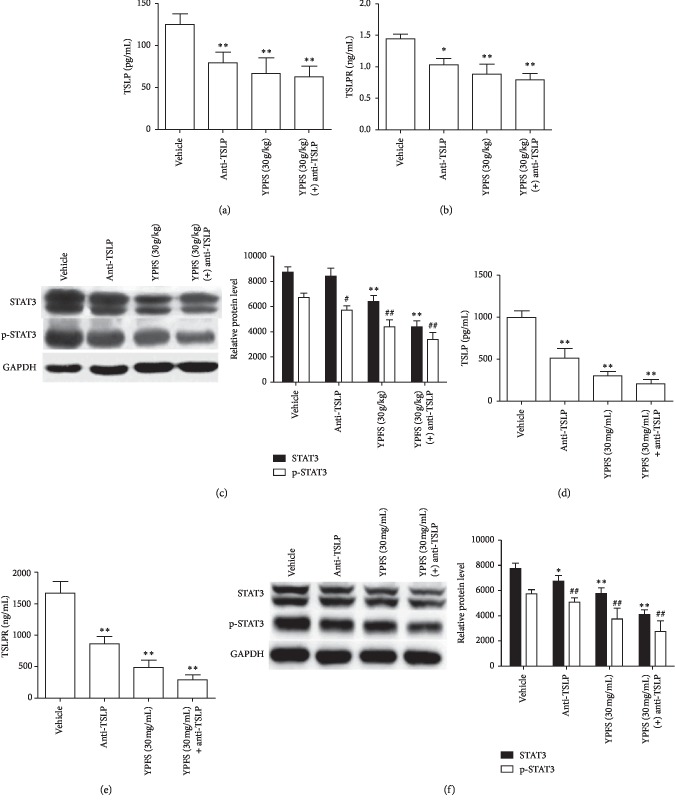
Anti-TSLP antibody can attenuate the inhibitory effect of YPFS on the signal pathways of TSLP/STAT3 in HCC. (a) Expression levels of TSLP in tumor tissues treated with the anti-TSLP antibody and indicated concentration of YPFS (30 g/kg) mice were measured by ELISA. (b) Expression levels of TSLPR in tumor tissues treated with the anti-TSLP antibody and indicated concentration of YPFS (30 g/kg) mice were measured by ELISA. (c) Western blot assays the expression of STAT3 and p-STAT3 in tumor tissues treated with the anti-TSLP antibody and indicated concentration of YPFS (30 g/kg). (d) ELISA analyzed the expression of TSLP in Hepa1-6 cell of the anti-TSLP antibody and indicated concentration of YPFS (30 mg/mL) treatment. (e) ELISA analyzed the expression of TSLPR in Hepa1-6 cell of the anti-TSLP antibody and indicated concentration of YPFS (30 mg/mL) treatment. (f) The expression of STAT3 and p-STAT3 in Hepa1-6 cell of the anti-TSLP antibody and indicated concentration of YPFS (30 mg/mL) treatment detected by western blot. Quantification of western blot analysis of p-STAT3 and STAT3 levels normalized by the levels of GAPDH. Data are presented as the mean ± standard error of the mean. ^*∗*^*P* < 0.05, ^*∗∗*^*P* < 0.01, ^#^*P* < 0.05, ^##^*P* < 0.01 compared with the vehicle group.

To probe into the molecular mechanism that YPFS inhibited the tumor angiogenesis through affecting the activation of TSLP, we examined the expression of TSLP/TSLPR and STAT3/p-STAT3 under the treatment of the anti-TSLP antibody and YPFS in vivo and in vitro. We found the expression of TSLP/TSLPR and STAT3/p-STAT3 in tumor tissues treated with the anti-TSLP antibody was significantly decreased compared with the vehicle group. However, when the anti-TSLP antibody treated along with YPFS, it did not cause further significant inhibition compared with group treated with YPFS only (*P* < 0.05 and *P* < 0.01; Figures 5(a)–5(c)). Then, we also detected the expression change of TSLP/TSLPR and STAT3/p-STAT3 in Hepa1-6 cell, and we found the results are in accordance with the result in vivo (*P* < 0.05 and *P* < 0.01; Figures 5(d)–5(f)). These results confirmed that YPFS suppressed angiogenesis through inhibiting the activation of TSLP/STAT3 signaling pathway.

### 3.6. Effect of YPFS on TSLP-Induced Vascularization of HUVEC

**Figure 6:**
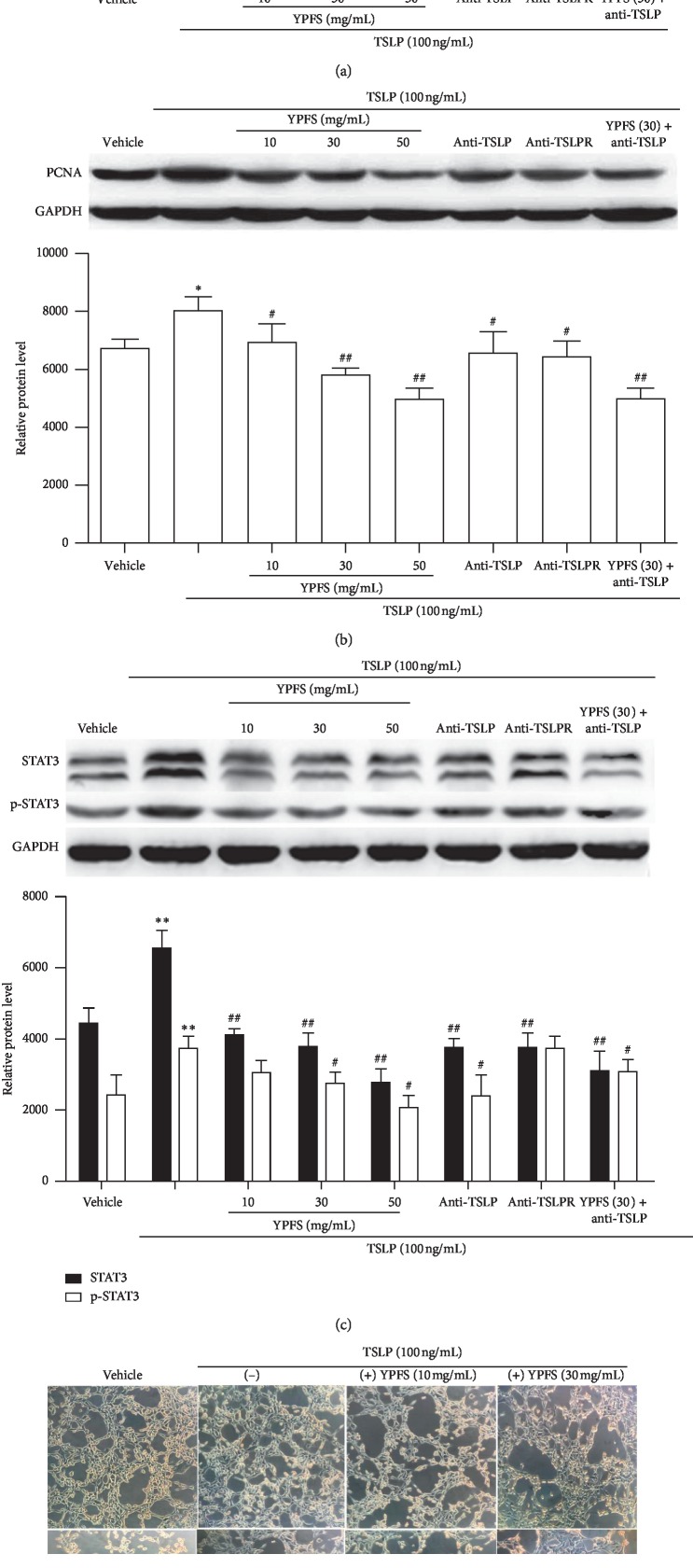
Effect of YPFS on TSLP-induced vascularization of HUVEC. (a) MTT assays with HUVEC cells induced by TSLP (100 ng/mL) combined with the antibody of anti-TSLP, anti-TSLPR and indicated concentration of YPFS (10, 30, 50 mg/mL). (b) The expression of PCNA in HUVEC treated with TSLP (100 ng/mL), the anti-TSLP and anti-TSLPR antibody and indicated concentration of YPFS (10, 30, and 50 mg/mL) detected by western blot. (c) The expression of STAT3 and p-STAT3 in HUVEC treated with TSLP (100 ng/mL), the anti-body of anti-TSLP, anti-TSLPR, and indicated concentration of YPFS (10, 30, and 50 mg/mL) detected by western blot. (d) HUVEC tube formation treated with TSLP (100 ng/mL), the anti-TSLP and anti-TSLPR antibody, and indicated concentration of YPFS (10, 30, and 50 mg/mL) (100x magnification). Quantification of western blot analysis of p-STAT3 and STAT3 levels normalized by the levels of GAPDH. Data are presented as the mean ± standard error of the mean. ^*∗*^*P* < 0.05, ^*∗∗*^*P* < 0.01, ^#^*P* < 0.05, ^##^*P* < 0.01 compared with the vehicle group.

To further examine whether the anti-angiogenesis activity of YPFS might be involved in the inhibition of the TSLP/STAT3 signaling pathway, we evaluated the effects of YPFS on the HUVEC induced by TSLP. The cell activity of HUVEC induced by TSLP was increased obviously, but the YPFS could inhibit the effect in a dose-dependent manner; meanwhile, the anti-TSLP and anti-TSLPR antibody could also inhibit this effect (*P* < 0.05 and *P* < 0.01; Figure 5(a)). The PCNA protein is a cellular marker for proliferation, then we investigated the expression levels of PCNA, the results showed that the expression changes of PCNA were consistent with the changes of the cell activity in HUVEC (*P* < 0.05 and *P* < 0.01; Figure 5(b)). These results indicated that YPFS has a remarkable inhibition on cell proliferation of HUVEC induced by TSLP. To confirm whether the anti-angiogenesis efficiency of YPFS was also mediated by blocking the TSLP/STAT3 signaling pathway in HUVEC, we investigated the expression levels of total STAT3 and the phosphorylated STAT3 through western blotting. The results showed that the expression levels of STAT3 and p-STAT3 was increased obviously, but the YPFS could inhibit the effect in a dose-dependent manner; the anti-TSLP and anti-TSLPR antibody could also inhibit this effect (*P* < 0.05 and *P* < 0.01; Figure 6(c)). Then we detected the tube formation of HUVEC; the results showed that the level of tube formation was consistent with the expression changes of STAT3 and p-STAT3 (Figure 6(d)). These results suggested that YPFS exerted anti-angiogenesis efficiency associated with the blockage of the TSLP/STAT3 signaling pathway and might be a promising anti-angiogenesis agent for the prevention of HCC.

## 4. Discussion

YPFS is extensively used as an immunomodulatory Chinese medicine for the treatment of immune system diseases. YPFS has a certain effect on Th2-type cytokines such as (IL-4, IL-6, IL-10, and IL-17) and Th1 type cytokines such as (IFN-*γ* and tumor necrosis factor-*α*), and YPFS mainly exerts pharmacological effects such as immune regulation and anti-allergy and anti-inflammatory by acting on these cytokines. Studies have shown that YPFS can inhibit the secretion of IFN-*γ* and IL-4, and affect the total number of T cells, which is reflected by the decrease of Th1 and Th2 cytokine level to mediate immunodeficiency disease [33, 34]. Chronic inflammation associated with Th2 cell polarizations can stimulate cancer progression [13]. Our previous research has shown that YPFS has a certain anti-tumor effect on the Hepa1-6-implanted liver cancer mice in a dose-effect by improving the immunosuppressive state of the liver cancer microenvironment. A novel finding of the present study was that YPFS could inhibit the angiogenesis and VEGF expression in tumor tissues of HCC-bearing mice, and reduce the secretion of VEGF in Hepa1-6. Further experiments showed that these effects of YPFS are regulated by inhibiting the TSLP/STAT3 pathways; meanwhile, the combination of anti-TSLP antibody can attenuate the effect. At the same time, it was also demonstrated that YPFS inhibited cell activity and the TSLP induced tube formation of HUVEC through the TSLP/STAT3 signaling pathway. Altogether, these results of the present study suggested that the anti-angiogenesis effect of YPFS participates in its suppression of tumor growth through the TSLP/STAT3 signaling pathway.

Angiogenesis is a necessary condition for the growth and metastasis of invasive tumors and an important link in the control of tumor progression [35]. The blood vessels of a solid tumor may actually be mosaic blood vessels composed of endothelial cells and tumor cells [36]. This interweaving causes a large number of tumor cells to fall into the blood vessels, which may contribute to the presence of circulating blood cells in peripheral blood of patients with malignant tumors [37]. Aberrant angiogenesis, especially solid tumors, plays a crucial role in tumor growth and metastasis. Proliferating tumor cells secrete large amounts of pro-angiogenic factors to activate tumor angiogenesis, providing oxygen and nutrients to tumor cells [38]. VEGF is a decisive manager of vascular permeability, promoting endothelial cell proliferation, migration, and tube formation; blocking VEGF leads to degradation of vascular network, and finally inhibiting the tumor growth and metastasis [39]. Many Chinese herbal ingredients have anti-angiogenic effects on VEGF-induced angiogenesis [40]. In the current study, we found the expression of VEGF in tumor tissues was markedly increased during tumor progression, but it was significantly decreased by YPFS administration. Meanwhile, the similar results occurred in Hepa1-6 cell. MVD may be involved in the capacity of tumor tissue to induce angiogenesis, and CD34 is considered to be a sensitive and well-defined marker of hepatic microvessels [30]. In this study, the expression of CD34 in microvessels was used as a surrogate marker to detect the expression of MVD in tissues. The results show that MVD in tumor tissues was significantly decreased by treatment with YPFS in a dose-dependent manner. It is indicated that YPFS can reduce the level of VEGF and angiogenesis in liver cancer tumors. Endothelial cell proliferation, migration, and capillary formation are critical events in the process of angiogenesis [41]. In this study, we showed that YPFS could effectively suppress the cell activity of TSLP-induced HUVEC in a dose-dependent manner. PCNA is an important indicator reflecting cell proliferation; we observed that PCNA-labeled cells in the TSLP-induced HUVEC were significantly decreased in the YPFS-treated groups. Meanwhile, the tube formation assay was used to mimic tumor angiogenesis in HUVEC in vitro, and the study indicated that the number of branches in the group treated with YPFS was smaller than that in the control group. Overall, these results suggest that YPFS may have potential anti-angiogenic activity in HCC.

TSLP, an IL-7-like inflammatory cytokine, is often associated with the induction of Th2-type allergic responses, which is expressed in cancers including human carcinomas such as breast cancer and melanoma. Based on the cross-talk between Th2 inflammation and cancers, a recent study shows that tumor progression is promoted by Th2-differentiated CD4^+^ T cells in response to TSLP [42]. The study suggests that TSLP plays an important role in cancer cell, and the inactivation of TSLP almost completely eliminates cancer progression and lung metastasis. TSLP mainly sends signals through TSLP receptors on CD4^+^ T cells to promote Th2-immune reaction abnormalities and produce immunosuppressive factors such as IL-10 and IL-13 [43]. TSLP has an important role in promoting the growth of vascular endothelial cells and angiogenesis, which could further promote the development and progression of cervical cancer [14]. Upon TSLP-dependent dimerization, regulates target gene expression via the JAK/STAT signaling pathway with the participation of signal transducers of STAT3, STAT5 and STAT1 [15]. Signaling through STAT3 increases the expression of many genes involved in cancer cell proliferation, survival, migration, invasion, and angiogenesis. The STAT3 transcription factor induces the expression of matrix metalloproteinase 2 (MMP-2), MMP-9, and epithelial-mesenchymal transition-related genes in promoting cancer cell invasion and metastasis. STAT3 activation occurs upon phosphorylation of Tyr705, leading to dimerization and translocation from the cytoplasm to the nucleus [44]. Persistent activation of STAT3, in addition to tumor cell proliferation and differentiation and metastasis, promotes the expression of related immunosuppressive factors, which causes immunosuppression of the tumor microenvironment [45]. In our study that YPFS inhibit the angiogenesis by decreasing the expression of TSLP/STAT3, which is in accordance with the improvement of immunosuppression of the tumor microenvironment. We speculate that the reduction of TSLP production induced by YPFS might contribute, at least partly, to the inhibition of the STAT3. To further verify whether TSLP/STAT3 signaling pathway participates in the anti-angiogenesis effect of YPFS, we used the anti-TSLP antibody and found it could attenuate the inhibitory effect of YPFS, which further confirmed YPFS through TSLP/STAT3 signaling pathway exerts the effect of anti-angiogenesis. The activation of STAT3 is indispensable in the process of tube formation, and blocking the activity of STAT3 can inhibit the migration and microvascular production of tumor vascular endothelial cells. The result that YPFS inhibit the TSLP induced tube formation of HUVEC through regulating the expression of STAT3, which proved STAT3 played a significant role in the angiogenesis of vascular endothelial cells.

The study demonstrated that STAT3 is a direct transcriptional activator of VEGF gene. In vivo inhibition of STAT3 activation by a negative mutant of STAT3 can significantly inhibit VEGF expression and angiogenesis, thereby limiting tumor growth and metastasis [46]. The sustained activation of STAT3 can induce a large amount of VEGF secretion from tumor cells and then bind to endothelial cell surface receptors to induce proliferation and activation of endothelial cells, thereby, promoting tumor angiogenesis and invasion and metastasis [47]. It has also been found that the regulation of VEGF expression by STAT3 plays a key role in the proliferation, migration, and microangiogenesis of HCC vascular endothelial cells, and suggests a “vicious circle” mechanism between STAT3 and VEGF [48]. Our study indicated that the down-regulated expression of VEGF was consistent with the STAT3, which demonstrated that STAT3 plays an important role in angiogenesis. However, further research should be performed to identify the results in the current study.

## 5. Conclusion

Taken together, the present study confirmed that YPFS inhibited angiogenesis and tumor growth in mice bearing HCC, which may be associated with the attenuation of the TSLP/STAT3 signaling pathway. Therefore, YPFS might serve as a novel candidate for the prevention of human HCC, and it should be investigated further. The study provided an experimental basis for new strategies for prolonging the survival of liver cancer patients and developing ideal therapeutic drug targets. The study may also contribute to uncovering the mechanisms and provide strong evidence for the use of such Chinese herbs in the treatment of HCC in China, and we hope that this research will pave the way for the future development of new Chinese complex prescriptions based on YPFS.

## Figures and Tables

**Table 1:** Mobile phase gradient elution programme.

Time (min)	Water (%)	Acetonitrile (%)
0	95	5
3	95	5
5	83	17
35	65	35

**Table 2:** Phytochemical composition of YPFS by HPLC analysis (1 g crude herbs/mL).

Number	The number of peak	Ingredient	Standard curve	*R* ^2^	Concentration (*μ*g)
1	5	prim-O-Glucosylcimifugin	*Y* = 13.273*X* + 88.432	0.9997	356.03
2	6	Calycosin-7-O-*β*-D-glycoside	*Y* = 14.362*X* + 93.928	0.9998	378.05
3	7	Cimifugin	*Y* = 18.209*X* + 64.563	0.9997	216.32
4	8	4-O-*β*-D-Glucosyl-5-O-methylvisamminol	*Y* = 11.508X + 79.576	0.9997	458.96
5	10	Psoralen	*Y* = 19.124*X* + 73.674	0.9997	82.06
6	11	Calycosin	*Y* = 23.541*X* + 54.335	0.9998	116.34
7	12	sec-O-Glucosylhamaudol	*Y* = 94.721*X* + 15.198	0.9997	165.18
8	13	Formononetin	*Y* = 15.122*X*+48.647	0.9998	91.14
9	15	Atractylon	*Y* = 13.1812*X* + 11.548	0.9997	51.40

## Data Availability

All data generated or analyzed during this study are included within the article.

## Abbreviations

YPFS: Yu ping feng san
HCC: Hepatocellular carcinoma
HPLC: High-performance liquid chromatography
MVD: Microvessel density
HUVEC: Human umbilical vein endothelial cell
VEGF: Vascular endothelial growth factor
TSLP: Thymic stromal lymphopoietin
AR: Astragalus membranaceus
AMR: Atractylodes macrocephala
SR: Saposhnikoviae radix
IHC: Immunohistochemistry.

## Supplementary Materials

Supplementary Materials Analyzed fifteen common peaks from the HPLC fingerprint of YPFS decoction. The peak of 5, 6, 7, 8, 10, 11, 12, 13, and 15 was prim-O-glucosylcimifugin, calycosin-7-O-*β*-D-glycoside, cimifugin, 4-O-*β*-D-glucosyl-5-O-methylvisamminol, psoralen, calycosin, sec-O-glucosylhamaudol, formononetin, and atractylon, respectively.
