# Supplementary material for: Yu Ping Feng San Exert Anti-Angiogenesis Effects through the Inhibition of TSLP-STAT3 Signaling Pathways in Hepatocellular Carcinoma
Source: Evid Based Complement Alternat Med. 2019 Oct 23;2019:1947156. doi: 10.1155/2019/1947156 (PMC6925680; doi:10.1155/2019/1947156)
Supplement: Supplementary Materials — Analyzed fifteen common peaks from the HPLC fingerprint of YPFS decoction. The peak of 5, 6, 7, 8, 10, 11, 12, 13, and 15 was prim-O-glucosylcimifugin, calycosin-7-O-β-D-glycoside, cimifugin, 4-O-β-D-glucosyl-5-O-methylvisamminol, psoralen, calycosin, sec-O-glucosylhamaudol, formononetin, and atractylon, respectively. [file 1947156.f1.pdf]

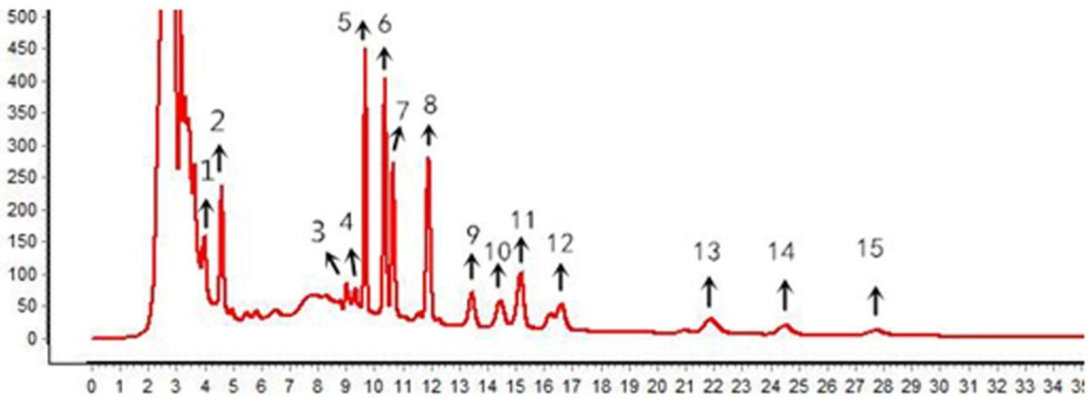

Fig 1. The HPLC fingerprint of YPFS decoction

Analyzed fifteen common peaks from the HPLC fingerprint of YPFS decoction. The peak of 5, 6, 7, 8, 10, 11, 12, 13, 15 was prim-O-glucosylcimifugin, calycosin-7-O-β-D-glycoside, cimifugin, 4-O-β-D-glucosyl-5-O-methylvisamminol, psoralen, calycosin, sec-O-glucosylhamaudol, formononetin and atractylon, respectively.
